# Role of filamin A in the pathogenesis of neuroendocrine tumors and adrenal cancer

**DOI:** 10.1530/EO-22-0055

**Published:** 2022-10-28

**Authors:** Donatella Treppiedi, Rosa Catalano, Federica Mangili, Giovanna Mantovani, Erika Peverelli

**Affiliations:** 1 Department of Clinical Sciences and Community Health, University of Milan, Milan, Italy; 2 Endocrinology Unit, Fondazione IRCCS Ca' Granda Ospedale Maggiore Policlinico, Milan, Italy

**Keywords:** FLNA, PitNETs, P-NETs, Pan-NETs, ACC

## Abstract

Cell cytoskeleton proteins are involved in tumor pathogenesis, progression and pharmacological resistance. Filamin A (FLNA) is a large actin-binding protein with both structural and scaffold functions implicated in a variety of cellular processes, including migration, cell adhesion, differentiation, proliferation and transcription. The role of FLNA in cancers has been studied in multiple types of tumors. FLNA plays a dual role in tumors, depending on its subcellular localization, post-translational modification (as phosphorylation at Ser2125) and interaction with binding partners. This review summarizes the experimental evidence showing the critical involvement of FLNA in the complex biology of endocrine tumors. Particularly, the role of FLNA in regulating expression and signaling of the main pharmacological targets in pituitary neuroendocrine tumors, pancreatic neuroendocrine tumors, pulmonary neuroendocrine tumors and adrenocortical carcinomas, with implications on responsiveness to currently used drugs in the treatment of these tumors, will be discussed.

## Introduction

Cell cytoskeleton proteins’ involvement in tumor pathogenesis, development and pharmacological resistance has been extensively explored in the last decades. Among these, Filamin A (FLNA), also known as actin-binding protein 280 (ABP 280), is a large cytoskeletal protein ubiquitously expressed in humans, whose role in cancers has been studied in a wide category of tumors (Shao *et al.* 2016, Zhou *et al.* 2021). Due to its crucial importance in cell migration and adhesion, FLNA was originally discovered as a tumor-promoting factor, closely involved in tumor invasion and metastasis. Ultimately, with increasing knowledge of its functions within the cell, FLNA is actually known to play a dual role in tumors, depending on the subcellular localization, and corresponding binding partners (Savoy & Ghosh 2013). Indeed, whether in the cytoplasm or the nucleus, FLNA facilitates the interaction of signaling molecules or transcription factors to either promote or prevent cancer. Moreover, this contradictory phenomenon is also related to FLNA post-translation modification and cleavage (Shao *et al*, 2016). Regarding FLNA expression, this was found upregulated in multiple types of tumors, including prostate, breast, lung, colon, neuroblastoma, squamous cell carcinoma and hepatic cholangiocarcinoma. Low FLNA expression levels were documented for renal cell carcinoma (Sun *et al.* 2014a), gastric cancer (Sun *et al.* 2014b), colorectal adenocarcinoma (Tian *et al.* 2015) and nasopharyngeal cancer (Sun *et al.* 2014c). In some instances, FLNA expression was found significantly correlated with lymph node metastasis, vascular or neural invasion, clinic stage, histological grade of tumor and average overall survival rate of patients (Shao *et al.* 2016).

The present review comprehensively summarizes available data on the role of FLNA in the cell biology of endocrine tumors, with particular attention to secreting pituitary neuroendocrine tumors (PitNETs), pancreatic neuroendocrine tumors (Pan-NETs), pulmonary neuroendocrine tumors (P-NETs) and adrenocortical carcinomas (ACC).

## FLNA: structure and function

FLNA is a ubiquitously expressed homodimeric actin-binding protein of 280 kDa encoded by a gene located on chromosome X (Hartwig & Stossel 1975). FLNA is a member of the FLNs family that includes other two homologous proteins named FLNB and C, encoded by genes located on chromosome 3 and 7, respectively. FLNB is also ubiquitous but less expressed than FLNA (Feng & Walsh 2004), whereas FLNC is predominantly expressed in cardiac, smooth and striated muscle (van der Flier & Sonnenberg 2001).

**Figure 1:**
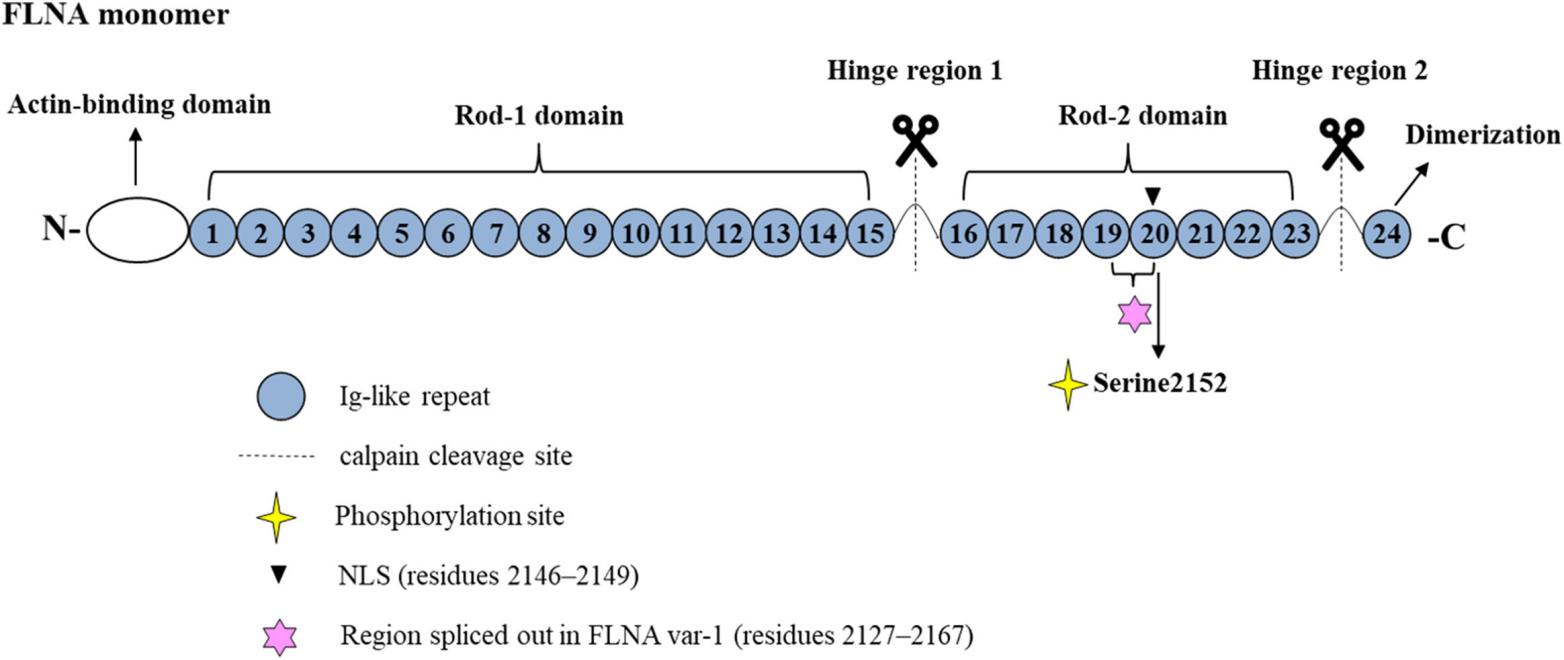
Schematic representation of an FLNA monomer. The structure of FLNA is schematically represented with the N-terminus domain containing the actin-binding site, followed by 24 immunoglobulin(Ig)-like repeats organized into rod domains: rod-1 (repeats 1–15), rod-2 (repeats 16–23) and repeat 24 that mediates homodimerization. Rod-1 and rod-2 are separated by a flexible hinge region (H1) and another hinge (H2) separates repeats 23 and 24. Both H1 and H2 are calpain cleavage sites. In repeat 20, the nuclear localization signal (NLS) and the phosphorylation site, serine 2152, are indicated. The region of 41 amino acids (repeats 19–20) deleted in the splice var-1 of FLNA is shown.

Structurally, the FLNA monomer consists of 2647 amino acids. The N-terminus domain contains the primary actin-binding site, followed by 24 immunoglobulin-like repeats of about 96 amino acid residues each organized into rods domains (rod-1 domain, repeats 1–15; rod-2 domains, repeats 16–23) and divided by a hinge region (H1). Another hinge region (H2) separates repeats 23 and 24, the latter representing the C-terminal self-association domain of FLNA that mediates FLNA homodimerization. Both H1 and H2 are susceptible to cleavage by calpain. The rod-1 domain contains a secondary actin-binding site of lower affinity, while the rod-2 domain is a region involved in the interactions with molecular partners (Fig. 1).

Thanks to its V-shaped flexible structure, the main function of FLNA is to crosslink actin filaments in order to confer membrane integrity, defend cells against mechanical stress and maintain cell shape (Hartwig & Stossel 1975, Stossel *et al.* 2001). A second structural function of FLNA is to physically connect transmembrane channels and receptors to the subcortical actin cytoskeleton. Moreover, the binding of FLNA with several intracellular proteins confers to FLNA the ability to work as a scaffold platform for different signaling pathways (Stossel *et al.* 2001, Nakamura *et al.* 2011). Indeed, FLNA is implicated in the regulation and integration of multiple cellular processes, including migration, cell adhesion, differentiation, proliferation and transcription.

Regarding FLNA mutations, in mice, a complete loss of FLNA expression causes embryonic lethality, cardiac malformations and skeletal defects (Hart *et al.* 2006). In humans, most hemizygous males harboring FLNA loss-of-function mutations die early during embryogenesis, while females present with a localized neuronal migration disorder known as periventricular nodular heterotopia or with other clinical disorders, named filaminopathies (Fox *et al.* 1998). FLNA gain-of-function mutations lead to congenital malformations (affecting brain, viscera and urogenital tract) and to skeletal dysplasia (Robertson 2005). FLNA variants can also manifest with seizures and cardiovascular and pulmonary findings (Robertson 2005).

FLNA activity is regulated by post-translational modifications. Particularly, among these, phosphorylation on the amino acid residue Ser2152 located in the repeat 20 is able to affect calpain-mediated proteolysis, binding affinity for molecular partners, conformation and cellular localization (Stossel *et al.* 2001, Nakamura *et al.* 2011) (Fig. 1). The cyclic adenosine monophosphate/protein kinase A (cAMP/PKA) pathway activation has been shown to induce FLNA phosphorylation in different cell systems (Chen & Stracher 1989, Jay & Stracher 1994). Moreover, a second PKA phosphorylation site is located in the N-terminal region of FLNA, probably involved in F-actin interaction (Jay & Stracher 1994). Besides PKA, other kinases such as PKC, CaM-kinase II, Pak1 (p21-activated kinase 1), RSK (ribosomal S6 kinase) and cyclin B1/Cdk1 may phosphorylate FLNA (reviewed inPeverelli *et al.* 2014b).

Another mechanism that modulates the FLNA function is represented by alternative splicing of FLNA mRNA. Indeed, FLNA splice variants may show different abilities to interact with molecular partners, as in the case of the FLNA splice variant-1 lacking 41 amino acids (residues 2127–2167) between the C-terminal part of repeat 19 and the N-terminal part of repeat 20 (Pentikäinen *et al.* 2011) (Fig. 1). FLNA splice variant-1 shows an enhanced FLNA/integrins binding compared with nonspliced FLNA (van der Flier *et al.* 2002).

## Role of FLNA in PitNETs

**Figure 2:**
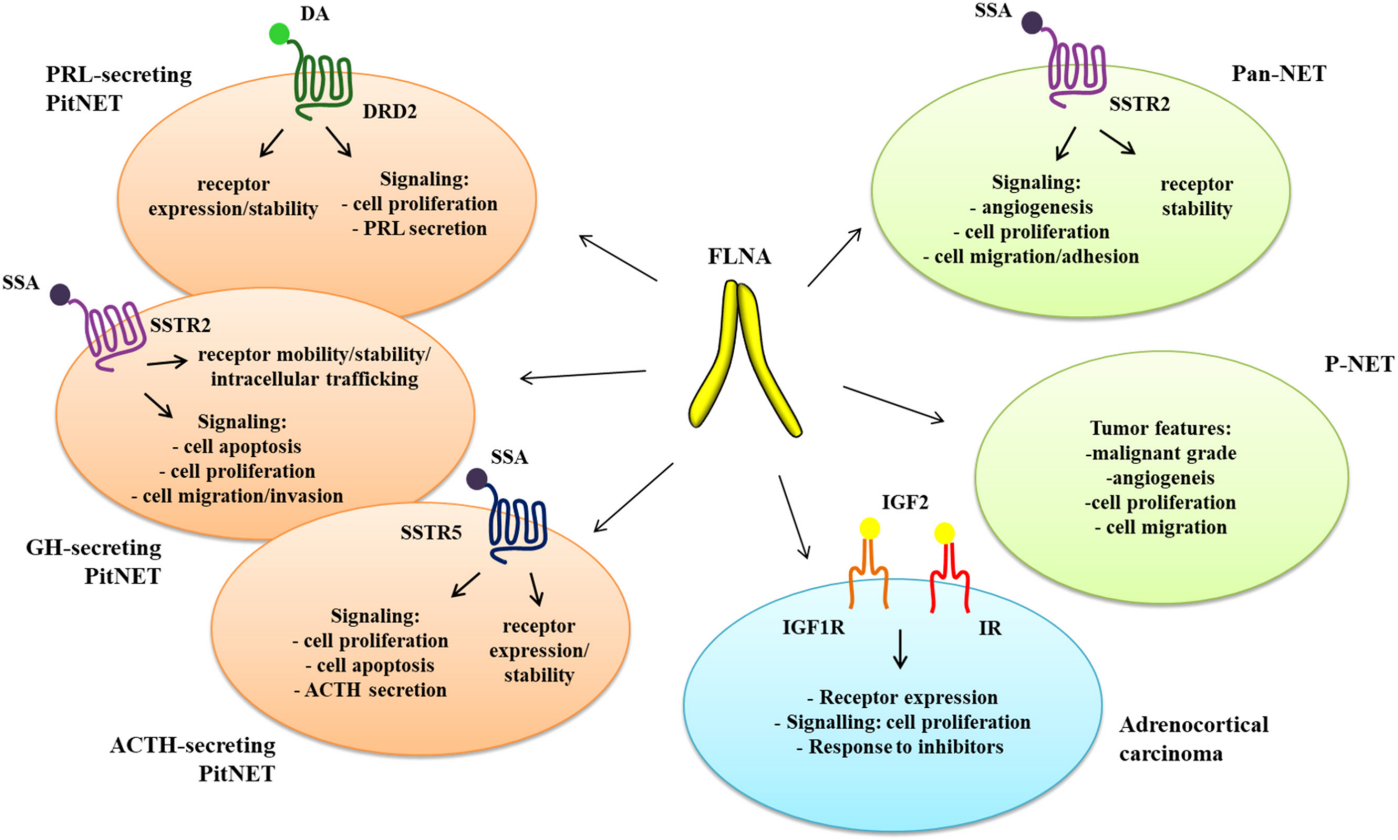
FLNA in endocrine tumors. The figure schematically represents the proposed models of FLNA involvement in regulating expression and functions of different receptors (DRD2, SSTR2, SSTR5, IGF1R, IR) specifically present in neuroendocrine tumors (PRL-, GH- and ACTH-secreting pituitary tumors, pancreatic and pulmonary neuroendocrine tumors, adrenocortical carcinomas).

In human tumors, FLNA may play opposite roles by differently regulating growth, invasion and metastasis (Shao *et al.* 2016). The relevance of FLNA in PitNETs relies on its ability to bind dopamine receptor type 2 (DRD2), somatostatin receptor type 2 (SSTR2) and 5 (SSTR5), the main pharmacology targets of prolactin (PRL)-, growth hormone (GH)- and adrenocorticotropic hormone (ACTH)-secreting PitNETs, respectively. In this scenario, FLNA plays a pivotal role in determining the antitumoral action of dopaminergic and somatostatinergic drugs since its proper function is essential for anchoring the receptors to the actin cytoskeleton and allowing the formation of multiprotein complexes with implications for receptors signal transduction, expression and intracellular trafficking, as discussed in details later and schematically depicted in Fig. 2.

### FLNA in PRL-secreting PitNETs

PRL-secreting tumors are the most frequent of all PitNETs subtypes and present with amenorrhea, galactorrhea and infertility in females and impotence or infertility in males. Pharmacological resistance of PRL-secreting tumors is commonly defined as failure to normalize PRL levels and to achieve tumor size reduction of at least 50% upon treatment with the dopaminergic drug cabergoline (Peverelli *et al.* 2021).

The first study establishing an involvement of FLNA in pituitary tumor pharmacological resistance was carried out in PRL-secreting tumors.

Indeed, in these tumors, resistance to cabergoline was associated with low or absent DRD2 expression along with low or absent FLNA protein expression (Peverelli *et al.* 2012). The *in vitro* characterization of the molecular mechanisms involved demonstrated a causal relationship between expression levels of FLNA and DRD2. Specifically, the reduction of hormone secretion and ERK1/2 phosphorylation observed in responsive primary cultured human PRL-secreting cells were impaired after FLNA silencing, while responsiveness to dopamine agonist (DA) was recovered in DA-resistant prolactinomas lacking FLNA after FLNA rescue (Peverelli *et al.* 2012). In MMQ cells, the rat cell line used as a model of PRL-secreting PitNETs, FLNA was necessary for DRD2 targeting to the plasma membrane and to protect DRD2 against lysosomal degradation (Peverelli *et al.* 2012) (Fig. 2), as observed for other receptors (Seck *et al.* 2003, Zhang & Breitwieser 2005, Thelin *et al.* 2007, Beekman *et al.* 2008), and for SSTR5, as further discussed in the ACTH-secreting PitNETs section. However, it remains to be established why FLNA expression is diminished in resistant tumors, since no alterations in the FLNA gene CpG island with the highest probability to have regulatory functions have been found, thus excluding epigenetic silencing (Peverelli *et al.* 2012).

It is worth mentioning that FLNA expression was correlated with DRD2 expression also in GH-secreting PitNETs (Coelho *et al.* 2019), suggesting that FLNA function in regulating DRD2 is conserved in different types of PitNETs.

FLNA functions are regulated by different mechanisms, including FLNA phosphorylation at residue Ser2152. This post-translational modification has been implicated in cell migration, integrin binding, calpain-mediated FLNA proteolysis and receptors recycling (Mantovani *et al.* 2019). It has been shown that cAMP/PKA pathway activation induced FLNA phosphorylation at S2152 in different cell systems including MMQ and AtT-20 cells (Mangili *et al.* 2021). At the same time, the DRD2 selective agonist, BIM53097, reduces the P-FLNA levels (Mangili *et al.* 2021). The effects of Ser2152 FLNA phosphorylation on DRD2 signal transduction were subsequently investigated by the use of a phosphomimetic S2152D FLNA mutant transiently transfected in MMQ and AtT-20 cells, which abrogated DRD2 agonist inhibitory effects (Mangili *et al.* 2021). Besides remarking the centrality of the cAMP pathway in PitNETs, these data revealed P-FLNA as a possible novel molecular biomarker for tumor responsiveness to DAs.

### FLNA in GH-secreting PitNETs

GH-secreting PitNETs are characterized by hypersecretion of GH by tumoral somatotroph cells and can cause either gigantism, if the tumor harbors during childhood, or acromegaly, if the tumor present later during adulthood. Particularly, acromegalic patients show clinical complications involving cardiovascular, respiratory and metabolic systems. The pharmacological therapy of GH-secreting PitNETs relies on the use of somatostatin analogs (SSAs) (octreotide and lanreotide) preferentially acting on SSTR2, the most representative SSTRs expressed on these tumors. However, only a subgroup of patients is adequately controlled by medical therapy (Peverelli *et al.* 2021). In GH-secreting tumoral cells, the role of FLNA in regulating SSTR2 dynamics and signaling has been extensively characterized, highlighting the crucial role played by this cytoskeleton protein in determining SSAs responsiveness. Moreover, the mechanisms modulating FLNA activity itself have been, at least in part, well documented.

In contrast to what was observed for DRD2 in prolactinomas (Peverelli *et al.* 2012), the protein expression of FLNA was not correlated with that of SST2 in a small group of GH-secreting PitNET tissues analyzed by Western blot (Peverelli *et al.* 2014a). A positive correlation has been documented in a cohort of patients by immunohistochemistry analysis only in not SSA-pretreated patients who were controlled with SSAs, strengthening an FLNA involvement in SSTR2 regulation and signaling (Coelho *et al.* 2019). The same study showed that in GH-secreting PitNETs, FLNA expression was correlated with SSTR5 expression (Coelho *et al.* 2019), but a contribution of FLNA in determining SSTR5-mediated effects has been further investigated in ACTH-secreting PitNETs, as described in the next section.

*In vitro* studies performed in different cell models have shown that FLNA is able to directly bind the first intracellular loop of SSTR2 with its repeats 19–20 (Najib *et al.* 2012), being this interaction crucial for controlling SSTR2 spatial arrangement and mobility at the cell surface and for orchestrating SST2 clusters formation and alignment along actin fibers (Treppiedi *et al.* 2018) (Fig. 2). In GH-secreting PitNET cells, FLNA is essential for SSTR2 internalization upon agonist incubation and recycling to the cell surface (Treppiedi *et al.* 2020). Besides its role as a scaffold platform that coordinates the intracellular trafficking of SSTR2, FLNA is implicated in its signal transduction. Indeed, FLNA alterations profoundly impacted on the SSTR2-mediated biological responses in both human GH-secreting PitNET primary cultured cells and GH3 cells. In the first case, FLNA knockdown abolished SSTR2-induced reduction of cyclin D1 and activation of caspase 3/7 required for reduction of cell proliferation and induction of cell apoptosis, respectively (Peverelli *et al.* 2014a). In GH3 cells, the overexpression of the dominant-negative mutant containing FLNA repeats mainly involved in scaffold function (repeats 21–24) abolished the SSTR2 effects on apoptosis and ERK1/2 inhibition (Peverelli *et al.* 2014a). In GH-secreting PitNETs, FLNA has been involved the anti-migratory and anti-invasive effects exerted by SSTR2 (Peverelli *et al.* 2018a) (Fig. 2). More specifically, the analysis of the pathway implicated in the regulation of cell motility by SSTR2 in GH3 cells revealed a central role of the actin-severing protein cofilin. Peverelli *et al.* showed that the selective activation of SSTR2 triggers recruitment of a protein complex that through FLNA scaffold region links cofilin to upstream kinases (RhoA/ROCK), leading to phosphorylation and inhibition of cofilin. Silencing experiments in GH3 cells showed that in the absence of FLNA, SSTR2 fails to co-immunoprecipitate cofilin and decrease cell invasion, providing a causal association between FLNA and SSTR2 anti-invasive signaling (Peverelli *et al.* 2018a).

In GH-secreting pituitary tumor cells, the activity of FLNA is influenced by cAMP/PKA-mediated phosphorylation at amino acid residue Ser2152, as observed in PRL- and ACTH-secreting cells (Mangili *et al.* 2021). FLNA phosphorylation was found to be upregulated or downregulated following forskolin or SSTR2 agonist incubation, respectively (Peverelli *et al.* 2018b). This post-translational modification has been shown to prevent the coupling of inhibitory G proteins to SSTR2, leading to the loss of anti-tumoral actions exerted by SSAs and acting as a dominant-negative mutant interfering with the function of endogenous FLNA. Indeed, in GH3 cells overexpressing the phosphomimetic S2152D FLNA mutant, SSAs failed to inhibit cell proliferation and migration, reduce cyclin D3, increase p27 and promote cell apoptosis. (Peverelli *et al.* 2018b).

All these pieces of evidence corroborate the idea that a loss of coupling of SSTR2 with downstream signaling partners determined by reduced FLNA expression or altered FLNA function might be at the basis of the pharmacological resistance to SSAs observed in GH-secreting PitNETs even if in presence of SSTR2.

### FLNA in ACTH-secreting PitNETs

ACTH-secreting PitNETs cause a rare and severe condition known as Cushing’s disease (CD). Patients with CD present with cardiovascular disease, diabetes, hypertension, osteoporosis, infections, and thromboembolic events (Pivonello *et al.* 2020), all findings deriving from the excess of ACTH-dependent cortisol release by the adrenal gland. Pasireotide is a second-generation multi-receptor SSA that preferentially binds to SSTR5 (Bruns *et al.* 2002), the subtype of SSTRs most abundantly expressed in ACTH-secreting PitNETs (Hofland *et al.* 2005, van der Hoek *et al.* 2005, Batista *et al.* 2006, Ibáñez-Costa *et al.* 2016). Pasireotide treatment achieves biochemical remission only in a subset of cases. Since* in vitro* pasireotide resistance was observed even in the presence of SSTR5, the mechanisms underlying resistance have been suggested to involve post-receptor alterations (Ibáñez-Costa *et al.* 2016).

An involvement of FLNA in the regulation of SSTR5 expression and signal transduction in ACTH-secreting PitNETs has been recently documented byTreppiedi *et al.* (2021). In their work, the authors pointed out a positive correlation between FLNA and SSTR5 expression levels in ACTH-secreting PitNETs tissues (Treppiedi *et al.* 2021). A similar association was previously reported in GH-secreting PitNETs (Coelho *et al.* 2019). Accordingly, FLNA silencing in primary cultured cells of human tumoral corticotrophs and At-T20 resulted in a reduction of SSTR5. As demonstrated in At-T20, FLNA prevents both lysosomal and proteosomal degradation of SSTR5 and its downregulation induced by prolonged pasireotide incubation (Treppiedi *et al.* 2021) (Fig. 2).

By *in situ* proximity ligation assay (PLA), the authors demonstrated a direct binding of FLNA to SSTR5 in AtT-20 cells (Treppiedi *et al.* 2021). Interestingly, pasireotide incubation promoted the formation of FLNA–SSTR5 complexes, in line with the role played by FLNA in pasireotide-mediated intracellular responses. The inhibitory actions of pasireotide were indeed abolished after FLNA silencing both in AtT-20 cells and in primary cultures of human tumoral corticotrophs *in vitro* sensitive to pasireotide (Treppiedi *et al.* 2021) (Fig. 2). Due to a possible mechanism of dilution of the partner proteins involved in the FLNA-regulated processes, a phenomenon already observed for other scaffold proteins (Burack & Shaw 2000), the overexpression of FLNA did not induce significant modification in SSTR5 protein expression or in pasireotide-mediated effects.

Cabergoline may represent a therapeutic option for ACTH-secreting PitNETs since these tumors also express high levels of DRD2. As previously discussed for PRL-secreting PitNETs in ‘FLNA in PRL-secreting PitNETs’, FLNA phosphorylation affected the ability of DRD2 to transduce the intracellular responses triggered by DA in AtT-20 cells (Mangili *et al.* 2021). Thus, phosphorylation might represent a novel regulatory mechanism that switches FLNA function from a scaffold platform enabling receptor signal transduction to a signal termination protein.

Summarizing, a key role of FLNA has been highlighted in ACTH-secreting PitNETs since it is directly involved in SSTR5 expression, intracellular signaling, degradation and downregulation. In addition, FLNA phosphorylation influenced DRD2 signaling and downstream actions of DA.

## FLNA in pancreatic neuroendocrine tumors

An involvement of the cytoskeleton protein FLNA in regulating SSTRs has been also demonstrated in Pan-NETs (Fig. 2). These tumors are a group of rare neoplasms arising either from the pancreatic islet cells of Langerhans or neuroendocrine cells that originate from the pancreatic ductal epithelium. The majority of Pan-NETs are hormonally nonfunctional and frequently sporadic. Surgery with complete resection is the only curative therapy for PNETs. SSAs are recommended for functional and well-differentiated tumors since Pan-NETs are characterized by the expression of SSTRs, particularly SSTR2 (Papotti *et al.* 2002, Reubi & Schonbrunn 2013), although they achieve control of symptoms and hormone secretion in 50–60% of patients with functioning NET and stabilize tumor mass in 30–50% of cases (Rinke *et al.* 2009, Öberg 2012, Caplin *et al.* 2014).

The first evidence of the importance of FLNA in human pancreatic tumor BON cell lines showed how FLNA was critical for SSTR2 stabilization and cell survival (Najib *et al.* 2012). Subsequently, the role of FLNA in SSTR2 expression and signaling, angiogenesis, cell adhesion and cell migration was explored in human Pan-NETs and in human pancreatic neuroendocrine cell line QGP1 (Vitali *et al.* 2016). In this work, the authors revealed that FLNA plays a pivotal role in SSTR2 stabilization at the membrane level, but it is not involved in SSTR2 expression in Pan-NETs, as in the case of GH-secreting PitNETs. They did not observe significant correlations between FLNA expression levels and either clinical presentation or pathological findings, as is the case for other tumors, such as the P-NETs further discussed in this review (Vitali *et al.* 2017). Experiments of FLNA silencing performed in QGP1 cells showed that in absence of FLNA the SSTR2 stability was impaired after long-term agonist incubation (Vitali *et al.* 2016). Moreover, the alteration of receptor stability resulting from FLNA silencing profoundly affected SSTR2 signaling in Pan-NETs: without FLNA, the selective SSTR2 agonist was unable to elicit the most relevant biological inhibitory responses mediated by SSAs on the intracellular cAMP, ERK 1/2 phosphorylation, cyclin D1 levels and VEGF secretion (Vitali *et al.* 2016). In addition, FLNA resulted critical in mediating the inhibition of two key events in tumor development, cell migration and the increase of cell adhesion, both induced by SSAs-activated SSTR2 (Vitali *et al.* 2016) (Fig. 2). Particularly, this is an important aspect to focus on giving that Pan-NETs are characterized by widely disseminated metastatic disease at diagnosis.

These data provide evidence for the role of the cytoskeleton in the control of neuroendocrine tumor progression triggered by SSTR2.

## FLNA in pulmonary neuroendocrine tumors

P-NETs represent a heterogeneous family of neoplasms ranging from well-differentiated typical carcinoids, to the intermediate-grade atypical carcinoids, to the highly malignant small-cell lung cancers (Travis 2014). For these tumors, surgery remains the treatment of choice (Gridelli *et al.* 2013). As mentioned earlier, FLNA is involved in tumor progression in some types of cancer, influencing cell mobility and invasion (Savoy & Ghosh 2013, Yue *et al.* 2013). Its role in determining P-NETs aggressiveness has been investigated by Vitali *et al.* (Vitali *et al.* 2017). First, a high FLNA expression was associated with a malignant grade of P-NETs. Besides histopathologic characteristics of the tumors, FLNA expression was also correlated with clinical parameters, including age and gender (Fig. 2).

Regarding the molecular mechanisms involved, the authors showed that FLNA promoted P-NET cell proliferation and colony formation since the cytostatic effect of FLNA silencing corresponded to a decrease in cyclin D1 expression (Vitali *et al.* 2017). Moreover, they demonstrated that FLNA knock-down significantly reduced VEGF expression in primary cultured P-NET cells and H727 cells (Vitali *et al.* 2017), highlighting a key role of FLNA in promoting angiogenesis and bring these results in line with the positive relationship between FLNA and VEGF seen in patients with lung cancer (Uramoto *et al.* 2010). By discovering a novel molecular partner of FLNA, the small GTPase Rap-1, they also showed that FLNA increased cell migration and decreased cell adhesion by a Rap1-dependent pathway in H727 cells (Caron 2003, Vitali *et al.* 2017) (Fig. 2). In agreement, FLNA silencing decreased cell migration and increased cell adhesion (Vitali *et al.* 2017). These data seem in agreement with results obtained in GH3 cells showing that the overexpression of FLNA truncated mutants 19–20 and 21–24, in the absence of SSTR2 stimulation, significantly inhibited cell invasion (Peverelli *et al.* 2018b). It is possible that FLNA differently binds key mediators of cell adhesion and migration and specifically these FLNA fragments may act as inhibitors of cell invasion. Indeed, they can bind the integrin β cytoplasmic tail, competing with talin, a major integrin activator (Kiema *et al.* 2006) and FLNA repeats 19–21 are known to be required for cell spreading and initiation of cell migration (Baldassarre *et al.* 2009).

Overall, this evidence in P-NET cells suggests FLNA as a potent modulator of cellular activities essential for cancer progression and metastasis development. It is reasonable that FLNA may be considered not only as a valuable diagnostic and prognostic markers but also as a novel therapeutic target.

## FLNA in adrenocortical carcinomas

ACC is a rare endocrine tumor, originating from the adrenal cortex, with a poor prognosis and an incidence between 0.7 and 2 cases per million each year (Berruti *et al.* 2021). To date, the best curative options are complete tumor resection and treatment with mitotane (Fassnacht *et al.* 2018, Amodru *et al.* 2020). Insulin-like growth factor (IGF) is one of the most studied pathways of ACC given that the 80–90% of patients have overexpression of IGF2 (Gicquel *et al.* 1994, Giordano *et al.* 2003, Almeida *et al.* 2008, Peixoto Lira *et al.* 2016, De Martino *et al.* 2019), one of the IGF ligands, which supports tumor growth (Guillaud-Bataille *et al.* 2014).

Recently, the role of FLNA in regulating the IGF system in ACC has been demonstrated (Catalano *et al.* 2021). By both co-immunoprecipitation experiments and PLAs in human ACC cell line H295R, the authors found that FLNA interacts with the two main receptors of the IGF pathway, IGF1R and the insulin receptor (IR). Receptors activation mediated by IGF2 resulted in a modification of their interaction with FLNA, with an increase of FLNA–IGF1R complexes and a reduction of FLNA-IR complexes (Catalano *et al.* 2021). Moreover, the presence of FLNA differentially regulated receptors expression activated by IGF2. In detail, FLNA silencing induced an increase in IGF1R expression and had variable effects on IR expression with a reduction in ACC cell lines and unchanged expression in ACC primary culture cells. Interestingly, the role of FLNA as a repressor of the IGF signaling cascade that promotes cell proliferation was highlighted. In fact, FLNA knockdown selectively enhanced the IGF2 effect on cell proliferation and ERK phosphorylation, but no variation was found in apoptosis signaling (Catalano *et al.* 2021). These results suggest that FLNA in ACC is a specific repressor of the IGF2-induced signaling cascade that, through ERK activation, promotes cell proliferation (Fig. 2).

Moreover, FLNA was also involved in the response of both dual IGF1R/IR inhibitor linsitinib and the specific IGF1R inhibitor NVP-ADW742. Indeed, FLNA silencing in H295R cells increased cell responsiveness to both inhibitors. In particular, FLNA-silenced cells were responsive to lower inhibitor concentrations, and at a concentration of 1 μM, the inhibition was significantly greater in silenced than in control cells (Catalano *et al.* 2021).

It is worth noting that these data were confirmed by Western blot analysis performed on ACC tissue samples showing FLNA/IGF1R and FLNA/IR ratios inversely correlated with P-ERK/total-ERK ratio. This data suggested that low FLNA levels, in the presence of high IGF1R and/or IR amount, corresponded to an enhancement of IGF1R pathway in ACCs. Additionally, FLNA expression, although variable, was significantly lower in ACC compared to adrenocortical adenomas, suggesting that in the absence of FLNA, an increased expression of IGF1R associated with the loss of the FLNA regulatory activity on the ERK pathway might concur to promote IGF2-induced cell growth.

Overall, these data highlighted the role of FLNA in reducing IGF2 mitogenic effects and the efficiency of IGF1R inhibitors in ACCs.

## Conclusions

In conclusion, the role of FLNA has been explored in different endocrine tumors, from PitNETs to Pan-NETs and P-NETs to ACC. In all these types of tumoral cells, FLNA combines with a variety of proteins, through direct or indirect interaction to affect downstream target molecules, which are the junctions and intersections of multiple pathways. Thus, changes in FLNA expression or phosphorylation state strongly impact on intracellular signaling, affect cytoskeleton remodeling with consequent alterations in mechanisms of cell migration and invasion, and are correlated with the pharmacological resistance phenomenon, this latter especially related to dopaminergic agonists and somatostatinergic analogs currently used in clinical practice for PitNETs. In this scenario, further works are needed to clarify the molecular events responsible for alterations in FLNA expression as well as post-translational modifications. Moreover, future studies searching for possible involvement of other cytoskeleton proteins might provide other insights to elucidate the complex mechanisms involved in endocrine tumor pathogenesis, progression and pharmacological resistance.

The development of drugs able to target FLNA and stabilize its interaction with molecular partners could be a novel field of research toward a more personalized strategy for the treatment of these types of endocrine tumors for which curative options are still limited.

## Declaration of interest

The authors have no conflict of interest to declare.

## Funding

This work was supported by AIRC (Associazione Italiana Ricerca Cancro) grants to G.M. (IG 2017–20594) and to E.P (IG 2021-25920), Ricerca Corrente Funds from the Italian Ministry of Health and Progetti di Ricerca di Interesse Nazionale (PRIN) grant to E.P. (2017N8CK4K).
